# Prolonged multisectoral aid-driven reliance on health systems and governance post-conflict era in Somalia

**DOI:** 10.1186/s41182-025-00861-y

**Published:** 2026-01-08

**Authors:** Saadaq Adan Hussein, AbdulJalil Abdullahi Ali, Marian Muse Osman, Abdirahman Moallim Ibrahim, Rage Adem, Mohamed Mohamoud Hassan, Yahye Sheikh Abdulle Hassan, Abdirahman Aden Hussein, Mohamed Abdullahi Awale, Mohamed M. Ali Fuje, Rufai Mohamed Salad, Abdinur Hussein Mohamed, Khadar Hussein Mohamud, Abdinur Adan Hussein, Abdirahman Dahir Ahmed, Abdishakur Mohamed Mohamud, Mohamed Abdurahman Hashi, Hassan Ahmed Mohamed, Ayan Nur Ali, Mohamed Farah Yusuf Mohamud, Omar Mohamed Mohamud

**Affiliations:** 1 School of Postgraduate Studies, Mentorship Coordinator Department of School of Postgraduate, Benadir University, Hodan Benadir, Mogadishu, Somalia; 2 Social and Human Capital Development Pillar, Office of the Prime Minister, Federal Republic of Mogadishu, Mogadishu, Somalia; 3 Research and Policy Development, SOR Institute: Somalia Social Research, Mogadishu, Somalia; 4 Benadir Institute for Research and Development, Benadir University, Mogadishu, Somalia; 5 Somali National Institute of Health, Mogadishu, Somalia; 6 Faculty of Medicine and Surgery, Jazeera University, Mogadishu, Somalia; 7 Director Innovation Hub, Benadir University, Mogadishu, Somalia; 8 Office Rector at Benadir University, Mogadishu, Somalia; 9 Faculty of Medicine and Surgery, Jamahiriya University of Science and Technology, Mogadishu, Somalia; 10 Faculty of Medicine and Surgery, Benadir University, Mogadishu, Somalia; 11 Director, Inclusive Politics Pillar, Office of the Prime Minister Mogadishu, Federal Republic of Mogadishu, Mogadishu, Somalia; 12 Director Office Somali Development Research Institute (SODRI), Mogadishu, Somalia; 13 Faculty of Civil Engineering at Amoud University, Borama, Somalia; 14 Policy and Reporting Advisor, Inclusive Politics Pillar, Office of the Prime Minister of Mogadishu Somalia, Mogadishu, Somalia; 15 Health Management Information Systems, Ministry of Health, Mogadishu, Somalia; 16 Emergency Resuscitation, Mogadishu Somali Türkiye Training and Research Hospital, Mogadishu, Somalia; 17 CEO Tayo Institute for Research and Development, Mogadishu, Somalia

**Keywords:** Somalia, Health, System, International, Aid, Post-conflict, Recovery, Governance

## Abstract

**Introduction:**

The global development discourse primarily emphasizes the vital role of international aid in post-conflict health systems and governance. Somalia’s post-conflict health recovery has relied heavily on multisectoral aid that saved lives but entrenched parallel systems. While the national budget rose from ~ SOS 200 million (2015) to ~ SOS 1.3 billion (2025), the Ministry of Health’s share remained ≤ 7%, leaving sustainability and local ownership at risk. Recent funding cuts have reduced food, health, and WASH services, heightening disease and malnutrition risks. This review examines the long-term impacts of multisectoral aid on Somalia’s health system and governance, focusing on its effectiveness, sustainability, and unintended consequences.

**Method:**

We conducted a narrative review (1990–2024; final search April 25, 2025) across PubMed, Scopus, Web of Science, Google Scholar, and gray literature (WHO, UNICEF, World Bank, USAID, FMoH). Using SANRA guidance, two reviewers screened 221 records plus prior evidence; 334 studies/reports were synthesized via hybrid thematic coding (NVivo) across five domains: aid-driven system development; aid–governance interactions; consequences of dependency; comparative insights; and sustainability pathways.

**Results:**

Aid delivered tangible “fruits”: expanded immunization and MCH coverage; high 2024 delivery performance (health 95% with US$69.8 M spent; nutrition 95.5% with US$73.9 M); and total donor inflows of ~ US$721.9 M fully deposited. However, most funds flowed off-budget through vertical programs and parallel supply/data chains, fragmenting governance and dampening state capacity. Despite the health share peaking at 7% (2023) and stabilizing near 6.8% (2025), cuts in 2025 curtailed essential services, leaving millions more vulnerable. Comparative cases (Liberia, Sierra Leone vs. South Sudan, Afghanistan) show sustainability improves when pooled funding, government payroll integration, and PHC-first strategies are adopted.

**Conclusion:**

For Somalia to transition from aid dependency to sustainable health governance, a deliberate shift is needed by strengthening FMoH leadership, funding PHC, unifying systems, integrating staff into public payroll, and ensuring epidemic readiness.

## Introduction

Global development discourse primarily emphasizes the vital role of international aid in post-conflict health systems and governance [1]. In a multisectoral aid landscape, health gains hinge on governance that aligns ideas, institutions, and interests toward SDG goals [2]. Achieving universal health coverage a core SDG target likewise rests on resilient systems spanning prevention, promotion, and care, underpinned by multisectoral governance that broadens access, curbs disease, and engages communities [3]. The Conflict simultaneously inflates healthcare needs through violence-related injuries and heightened infectious-disease transmission while crippling service delivery by destroying facilities, driving health workers away, and obstructing patients’ safe passage to care [4]. Post-conflict decisions significantly affect global quality of life, necessitating theoretical reflection to understand potential tensions and opportunity costs due to practical constraints and resource scarcity [5]. Decentralization involves the transfer of authority and responsibility from central to subnational government levels, involving power-sharing arrangements and decision-making at different levels [6]. Somalia’s post-conflict recovery process highlights the benefits and limitations of multisectoral aid, highlighting its geopolitically strategic position and attracting global interest and external interventions [7, 8]. After gaining independence in 1960, the Republic of Somalia initially pursued centralized state-building efforts [9].

Since the early 1990s, the country has been gripped by a complex crisis marked by the collapse of the central government, protracted armed conflict, and pervasive lawlessness [10]. The fall of Siad Barre in 1991 created a power vacuum, triggering a brutal civil war and decades of institutional decay [11, 12]. This era entrenched deep political fragmentation, driven by a combination of internal clan divisions, economic disparities, and external influences, including Cold War geopolitics and regional rivalries [7]. Clan competition, resource scarcity, and Cold‑War rivalries combined to unravel state institutions [7]. The resulting collapse of state structures gave rise to governance challenges, including constitutional ambiguities, weak consensus-building mechanisms, unresolved issues with Mogadishu’s administration, and difficulties implementing fiscal federalism [11]. In response, transitional governing bodies were established, culminating in the formation of the Federal Government of Somalia in 2012 [10]. This marked a pivotal step toward political normalization, albeit within a deeply fragmented and contested federal framework. Rebuilding efforts in the early 2000s saw attempts to rebuild institutions, heavily reliant on international aid and diplomacy [13–15].

**Fig. 1:**
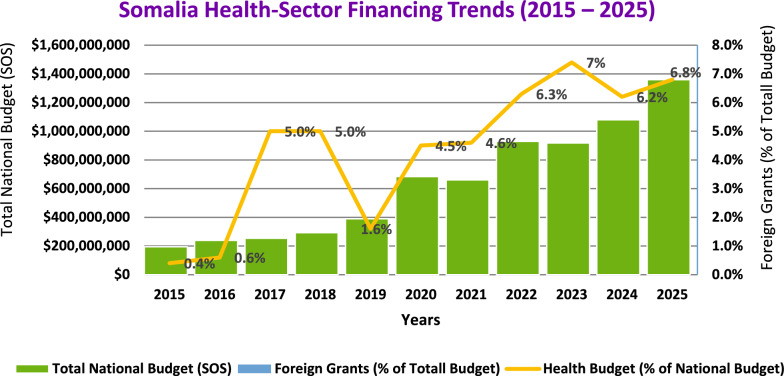
Analysis of health sector financing trends in Somalia (2015–2025) [16]

Somalia’s national budget grew significantly—from about SOS 200 million in 2015 to approximately SOS 1.3 billion by 2025. The health sector’s share of the budget also fluctuated: starting below 1% in 2015, rising to 5% in 2017–2018, dropping to 1.6% in 2019, then steadily increasing to a peak of 7% in 2023, before settling at around 6.8% in 2025 (Fig. 1) [16]. As of 31 December 2024, Somalia’s humanitarian fund had mobilized US $ 723.8 million, of which US $ 723.3 million had been utilized, leaving a closing balance of US $ 0.56 million and US $ 30.8 million still with implementing partners (Table 1). Donor inflows reached US $ 721.9 million, fully deposited, with Germany, Denmark, Sweden, Norway and Australia among the principal contributors (Table 2). These resources financed US $ 716 million in cluster-level actions, led by Water and Sanitation (US $ 136 million), Multi-Cluster Projects (US $ 106 million) and Food Assistance (US $ 104 million). Substantial allocations also supported Nutrition (US $ 76 million), Health (US $ 72 million), Protection (US $ 38 million), Enabling Programming (US $ 31 million), Livelihoods (US $ 29 million) and Education (US $ 24 million). Operational support for Camp Coordination and Management, Logistics and Direct Costs together totalled US $ 37 million, while multi-sector services for refugees received US $ 1.5 million (Table 3).

**Table 1:**
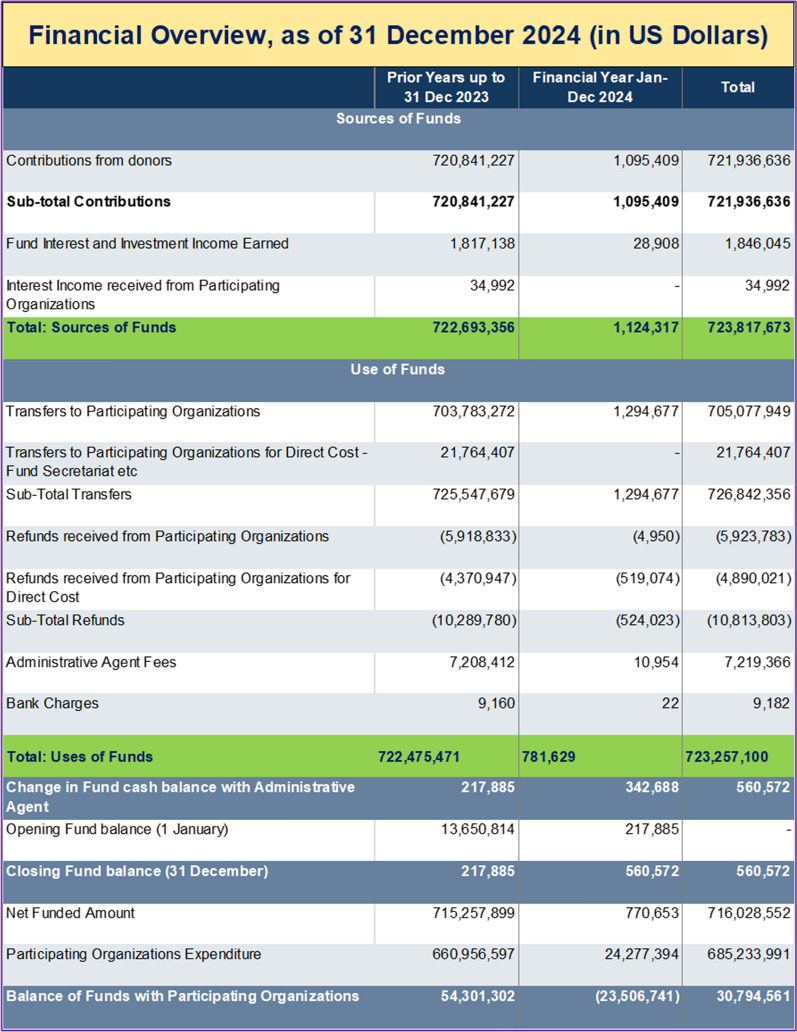
Financial overview Somalia of sources and uses of funds as of 31 December 2024 (US Dollars)

**Table 2:**
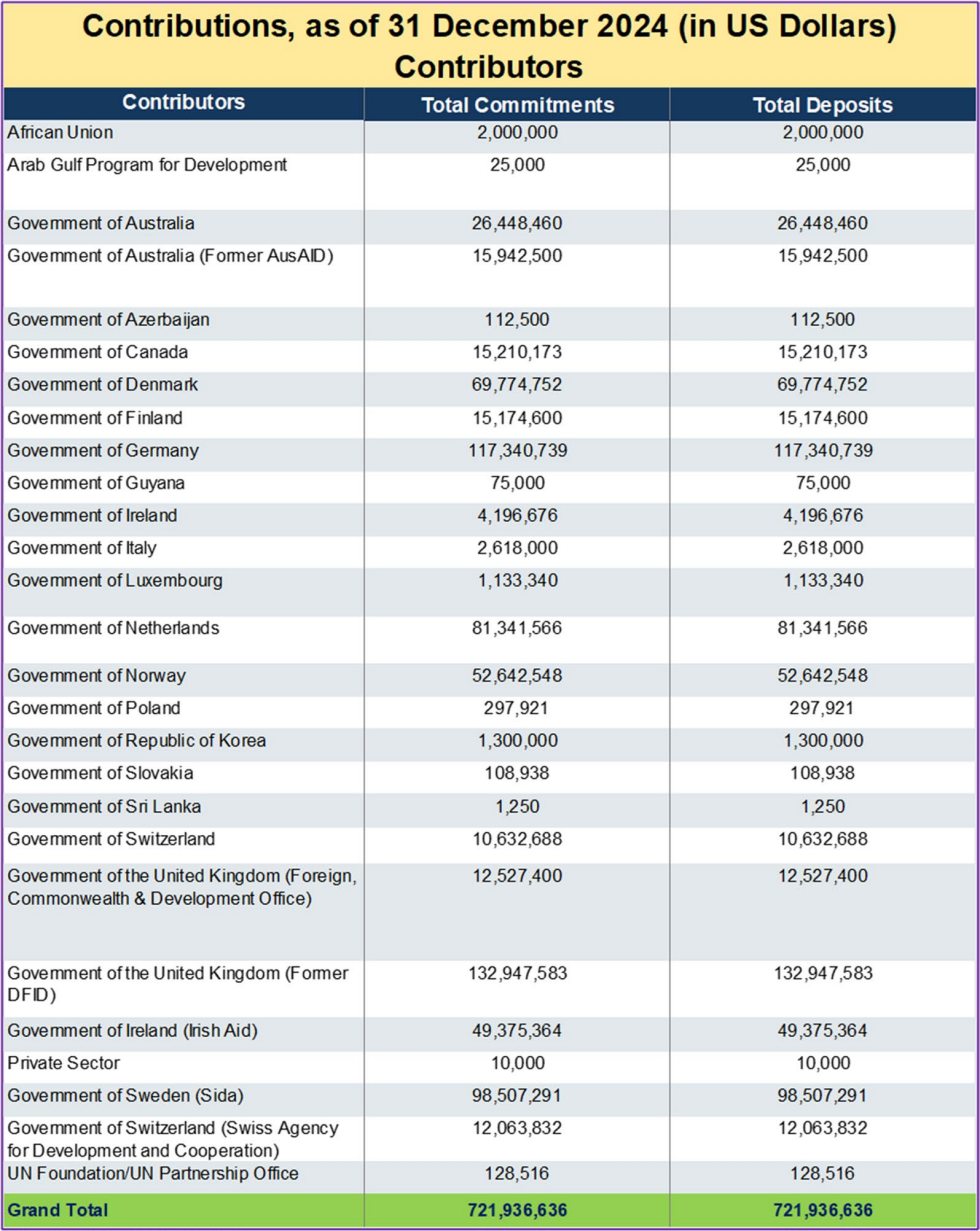
Donor contributions to Somalia as of 31 December 2024 (in US Dollars)

**Table 3:**
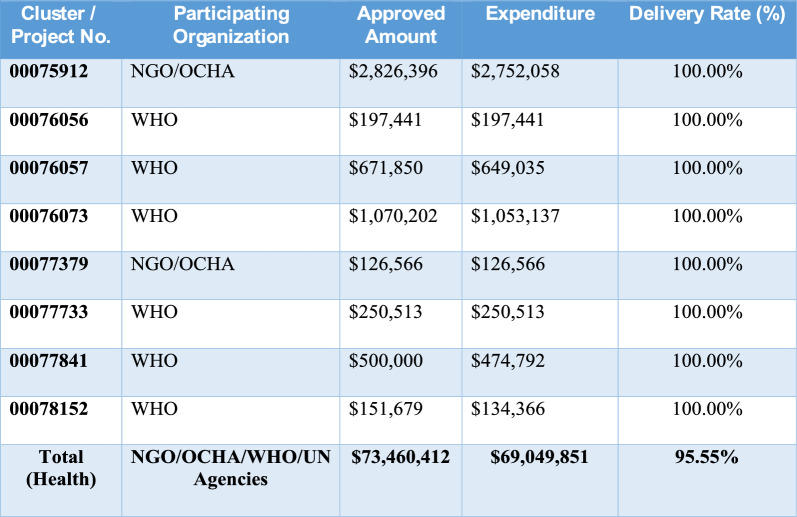
Health sector projects in Somalia: approved amounts, funding, expenditures, and delivery rates as of 31 December 2024

Across over 170 interventions, the health portfolio mobilized US $73.5 million, with a delivery rate of 95.6%, as 98% was disbursed and US $69 million spent. Most grants are closed at full utilization; only a few recent awards remain active, exceeding 95% delivery. About 100 nutrition projects obtained US $77.7 million in approvals, with US $76.5 million released and US $73 million expended, resulting in a 94.5% delivery rate. Most activities are closed and executed, with ongoing grants maintaining delivery levels above 94% [17]. The International aid has been central to Somalia’s post-conflict recovery, with multisectoral support targeting health, education, and governance. Health system reconstruction became a key focus, addressing critical gaps in infrastructure, services, and workforce capacity [18–21]. Despite Somalia’s health system is largely dominated by private providers, delivering around 80% of curative services and acting as referral hubs. Most care is financed out-of-pocket, while weak regulation, poor coordination, and limited accountability hinder equitable access [22].

This year 2025 funding cuts are forcing agencies in Somalia to scale back food, health, and water services, leaving over 2 million people at greater risk [23], have left hundreds of thousands without safe water, driving the spread of preventable diseases, over three million people risk losing vital aid [24], and faces soaring malnutrition as funding cuts shut over 300 nutrition centers worsening the crisis [25]. However, limited local ownership and sustainability remain concerns. The federal transition requires tailored strategies to strengthen institutions and mitigate ongoing instability [26]. This review examines the long-term impacts of multisectoral aid on Somalia’s health system and governance, focusing on its effectiveness, sustainability, and unintended consequences.

## Method

### Study design and area

Somalia, a low-income federal republic located in the Horn of Africa, shares borders with Ethiopia to the west, Kenya to the southwest, Djibouti to the northwest, and the Indian Ocean along its eastern coastline. Administratively, the country is divided into six federal member states and one additional administrative region [27] and has an estimated population of 18.1 million, with the majority residing in urban centers such as the capital city, Mogadishu [28].

Somalia’s rural regions face major challenges, including weak infrastructure and limited healthcare access. Health governance is divided between the Federal Ministry of Health and state-level ministries, while service delivery is largely driven by primary health posts and maternal and child health centers. Donor funding constitutes nearly half of total health expenditure, and health workforce density remains below the WHO-recommended threshold [18, 29].

This study employs a narrative review design to examine the prolonged reliance on multisectoral, aid-driven interventions in shaping Somalia’s health systems and governance in the post-conflict era. By identifying trends, challenges, and opportunities associated with aid dependency, this study provides critical insights into the current state and future direction of Somalia’s health system. It also highlights gaps in policy alignment, governance capacity, and sustainability, offering a foundation for evidence-based strategies toward a more resilient and self-sufficient health governance model.

**Fig. 2:**
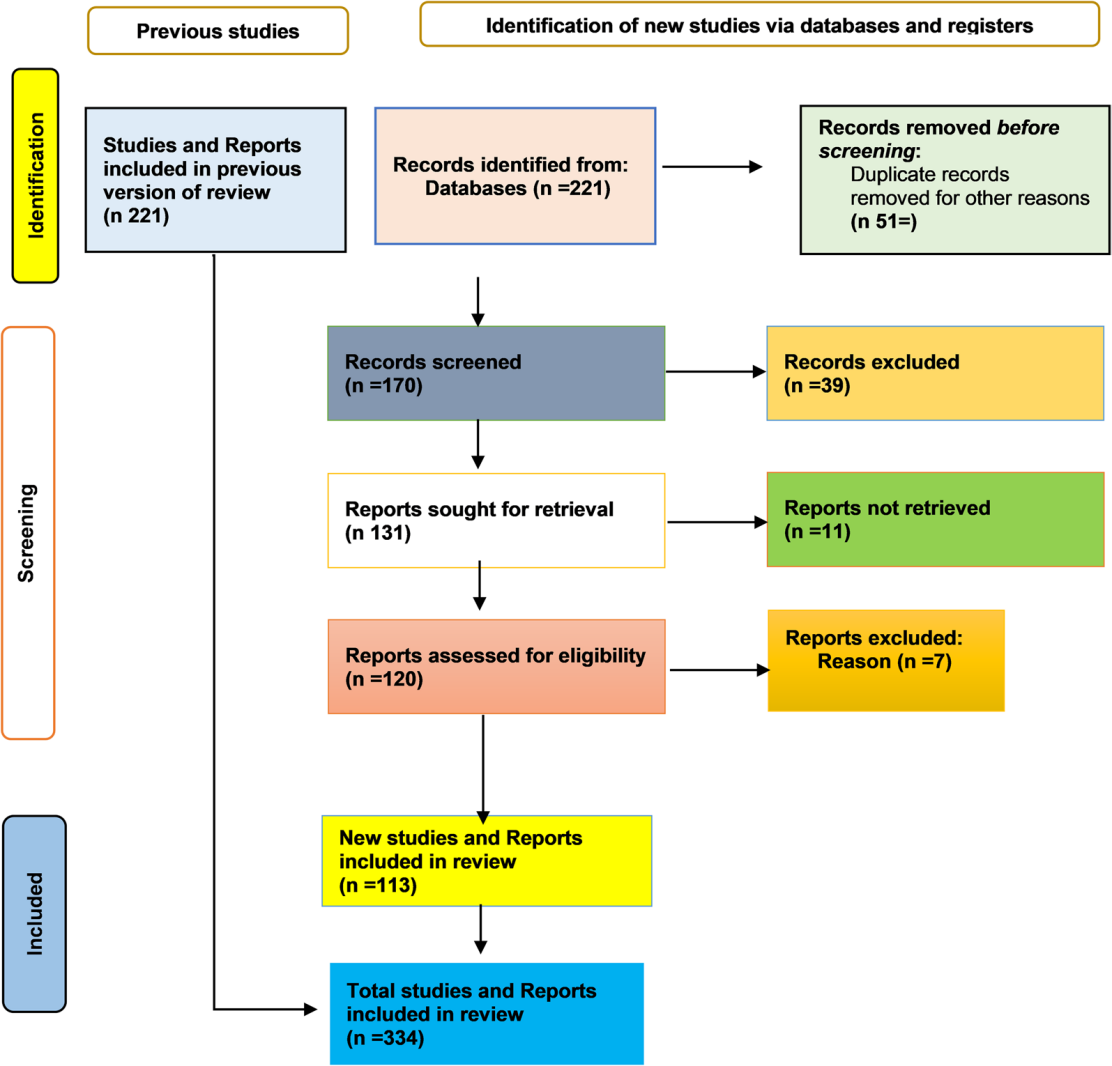
PRISMA flow diagram selection flowcharts

This study used a narrative review guided by PRISMA-SANRA (Scale for the Assessment of Narrative Review Articles) [30] was conducted on articles from PubMed, Scopus, Web of Science, and Google Scholar explored the impact of multisectoral aid interventions on Somalia’s health systems and governance post-conflict. A total of 221 records were identified, with 51 removed as duplicates or irrelevant. Of 170 records screened, 39 were excluded and 11 could not be retrieved. From 120 full-text reports assessed, 7 were excluded, leaving 113 new studies included. Combined with 221 previously included, the final review comprised 334 studies and reports (Fig. 2).

### Data sources, search strategy and data extraction process

A comprehensive literature search was conducted across multiple academic databases, including PubMed, Scopus, Web of Science, and Google Scholar. Searches were conducted November 2024–March 2025, with a final search on April 25, 2025. Data were coded in NVivo 12 using a hybrid approaching deductive coding. The review also incorporated grey literature from reputable international organizations such as the World Health Organization (WHO), UNICEF, the World Bank, USAID, and the Somali Ministry of Health. Policy documents, program evaluations, donor reports, and relevant NGO publications were included to capture the practical dimensions of aid implementation. The search covered literature from 1990 to 2024 using keywords such as: (Somalia OR “Horn of Africa”) AND (aid depend* OR “foreign aid” OR “multisectoral assistance” OR “development cooperation”) AND (“post-conflict recover*” OR govern* OR federalism OR “institutional capacity*” OR “aid effective*” OR sustainable* OR “donor coordination”). The data were coded inductively using NVivo 12, and after applying a six-step thematic analysis, they were synthesized into four key themes, and two reviewers independently handled screening and data extraction, resolving differences by consensus. Thematic analysis was then conducted to organize findings across the following domains: I. aid-driven health system development. II Aid–governance interactions. III. Consequences of prolonged aid dependency. IV. Global and regional comparative insights. V. Pathways toward sustainability.

### Inclusion and exclusion criteria

**Included:**

- Post-1990 Somali context, especially relating to health, governance, and aid
- Empirical studies, policy analyses, organizational reports, and reviews
- English-language publications dated between 1990 and 2024.

**Excluded:**

- Non-Somalia-specific literature or documents unrelated to governance/health sectors
- Opinion pieces lacking empirical basis
- Outdated, low-quality, or duplicative documents.

## Result: thematic body of the review

### Aid-driven health system development

#### Health system rebuilding post-conflict

Following the collapse of Somalia’s central government in 1991, the national health system deteriorated rapidly, leading to a vacuum in service provision, loss of infrastructure, and outmigration of health professionals. In the absence of a functioning state, the international aid community comprising United Nations agencies, bilateral donors, and non-governmental organizations (NGOs) stepped in to fill critical gaps, these actors-initiated humanitarian and emergency health programs, laying the foundation for what would become the aid-driven architecture of Somalia’s health system [31–34]. Over the past three decades, the process of health system rebuilding in Somalia has been heavily influenced by the priorities and operational modalities of external partners rather than by a national, long-term development framework. This external influence has led to a fragmented system shaped more by donor interests and funding cycles than by cohesive policy direction or domestic capacity [35, 36].

The re-emergence of governance structures, particularly the Federal Government and the Federal Ministry of Health and Human Services (FMoH) did not fundamentally shift this trajectory. Instead, the role of the state has remained largely that of a coordinator rather than a primary provider or steward of health services. As a result, donor funding continues to be the dominant driver of health sector development, often bypassing government systems through parallel channels [18, 37]. The bulk of service delivery, particularly in maternal and child health, nutrition, and communicable disease control, is still carried out by international actors operating in silos, with limited integration into the national health system.

#### Positive contributions of aid: service access, disease control, and MCH gains

Despite the concerns around sustainability and ownership, international aid has played a critical role in improving health indicators and expanding access to essential services in Somalia. Donor-funded programs have contributed significantly to increasing immunization coverage, reducing child mortality, and improving maternal health services in both urban and rural settings [38, 39]. For instance, the Expanded Programme on Immunization (EPI), supported by Gavi, UNICEF, and WHO, has reached millions of children, even in insecure and hard-to-reach areas [40–42]. Over 80% of donor health funding in Somalia is delivered through UN agencies, international NGOs, and the Red Cross/Red Crescent, bypassing government financial systems. In 2020 alone, 96.8% of these funds were managed off-budget and off-treasury, with NGOs receiving 61%, UN agencies 30%, and Somali government entities only 3% (Private Sector Partnerships in Health, 2021) [43].

These implementing partners operate through parallel structures maintaining independent systems for finance, procurement, human resources, and data management. As a result, medicine procurement, health worker salaries, and real-time service data remain siloed, outside of national platforms. The Federal and State Ministries of Health often access only aggregated reports, limiting their ability to govern or integrate systems effectively (Ministry of Health Somalia, 2022). While this arrangement expedites service delivery amid Somalia’s fragile context, it undermines national ownership and hampers efforts to build a unified, resilient health system [44].

Additionally, disease surveillance and outbreak response capacity particularly for cholera, measles, and polio—have been enhanced through Global Health Security Agenda initiatives and the Integrated Disease Surveillance and Response (IDSR) framework, supported by the Centers for Disease Control and Prevention (CDC) and WHO [45–47]. Malaria, HIV, and tuberculosis (TB) programs, largely financed through the Global Fund and international NGOs, have achieved measurable progress in reducing disease burden and improving case detection and treatment outcomes [48, 49].

Furthermore, the humanitarian health cluster system, coordinated by the World Health Organization, has enabled a coordinated emergency response during crises such as floods, droughts, and COVID-19 outbreaks. These efforts have helped avert catastrophic public health consequences in the context of chronic instability [50]. Multisectoral programs that integrate nutrition, WASH (water, sanitation, and hygiene), and reproductive health services have also demonstrated positive impacts on vulnerable populations, especially internally displaced persons (IDPs) [51, 52].

#### Persistent issues: vertical programs, parallel systems, and lack of integration

However, the architecture of aid delivery has often prioritized short-term, disease-specific interventions at the expense of long-term system strengthening. Most donor support is channeled through vertical programs that operate independently of national systems, focusing narrowly on specific health outcomes rather than strengthening health governance or infrastructure [53, 54]. These vertical interventions, while effective in achieving quick wins, have contributed to fragmentation, duplication of services, and inefficiencies in resource allocation [55].

Moreover, parallel systems established by donors and implementing partners have undermined government leadership and reduced incentives for domestic investment in the health sector. These systems often include independent supply chains, health information platforms, and workforce arrangements that bypass government oversight. As a result, coordination challenges persist, and the health system remains heavily reliant on external technical and financial assistance [53, 56, 57].

The absence of integrated primary health care (PHC) models and the limited institutionalization of donor-supported initiatives have further hindered the development of a cohesive and resilient national health system. Despite efforts by the FMoH to adopt frameworks like the Essential Package of Health Services (EPHS) and the Health Sector Strategic Plan (HSSP), implementation remains inconsistent due to funding gaps, security constraints, and limited technical capacity. In addition, the decentralization of governance to federal member states has complicated service delivery and accountability, with many states relying on ad hoc donor arrangements without a unified national approach [18, 58].

This prolonged dependency on donor-driven programs poses significant risks to the sustainability of Somalia’s health gains. As global aid landscapes shift and funding priorities evolve, the need for transition strategies that strengthen local ownership, build institutional capacity, and promote integration of services becomes increasingly urgent.

### Aid and governance interactions

#### How aid influenced health governance, planning, and leadership

In Somalia’s post-conflict landscape, the influx of multisectoral aid has had a profound influence on the development and function of national health governance structures. While aid has undeniably played a vital role in reviving service delivery and re-establishing a health presence in both urban and rural areas, it has simultaneously shaped and in many cases constrained—the country’s capacity to lead, plan, and manage its own health priorities [21, 59]. Aid inflows often come with predetermined priorities, timelines, and funding conditionalities that do not always align with Somalia’s long-term public health needs or national development strategies. For instance, donor emphasis on specific vertical programs such as immunization, maternal and child health (MCH), or HIV and TB control has frequently overshadowed broader systemic investments in health infrastructure, workforce development, and primary care integration [60]. These programmatic silos, while effective in achieving immediate targets, have often sidelined national leadership in planning and decision-making processes. The Federal Ministry of Health and Human Services (FMoH) has made strides in developing policy frameworks, such as the Health Sector Strategic Plan (HSSP III) and the Essential Package of Health Services (EPHS). However, the ability of these policies to guide implementation is frequently undercut by donor fragmentation and the limited alignment of partner programs with government priorities [61]. Although some development partners collaborate closely with the Ministry, many continue to operate independently, often through international NGOs and UN agencies that report outside formal government structures. This bypassing of national systems undermines local leadership and restricts the Ministry’s ability to develop institutional capacity and long-term resilience.

#### Weak capacity versus donor-driven priorities

A major challenge in Somalia’s health governance is the tension between the government’s weak capacity and donor influence on sectoral priorities. In the absence of strong state structures especially in the earlier years of transition—donors naturally filled the governance vacuum by establishing parallel systems and fast-tracking service delivery through international partners. While this model ensured immediate coverage of lifesaving services, it unintentionally institutionalized dependency and eroded the motivation for domestic investment [18]. Donors, while generally well-intentioned, often impose programmatic and budgetary priorities based on their own strategic interests, sometimes with limited contextual grounding. These externally driven approaches can marginalize national health priorities, especially those that require long-term investment, such as non-communicable diseases (NCDs), mental health, or health information system strengthening [18, 53]. Moreover, aid fragmentation where different donors fund similar projects through multiple implementing partners have contributed to resource duplication and program inefficiencies.

When several donors fund similar Somali health projects through separate UN agencies or NGOs, each imposes its own budgeting rules, reporting cycles and procurement channels, so staff spend more time meeting multiple compliance requirements, parallel supply chains bid against one another, and services cluster in the same “easier-to-reach” districts while others are missed classic symptoms of aid fragmentation that raise overheads and leave coverage gaps despite larger headline budgets [62]. As a result, the Ministry of Health frequently struggles to enforce standards, monitor performance, or maintain continuity across projects. This dynamic creates a vicious cycle: the government is perceived as weak, donors take on more responsibility, and the state is further marginalized. Without intentional efforts to invest in government capacity technical, managerial, and financial this cycle perpetuates a system in which national ownership remains elusive.

#### Role of coordination mechanisms: health sector coordination forums

Recognizing the need to harmonize efforts and enhance government leadership, Somalia and its partners have gradually established coordination mechanisms to bridge the governance gap. Chief among these is the Health Sector Coordination Forums (HSCFs), which are led by the FMoH and include representatives from UN agencies, NGOs, donors, and subnational health authorities. These platforms provide a space to discuss priorities, review progress, and align efforts with national health strategies [37]. At their best, HSCFs foster transparency, build trust between stakeholders, and serve as incubators for joint planning and resource mapping. They are also essential for policy endorsement, as they enable the Ministry to review and validate externally developed programs. However, the functionality of these forums is not consistent across regions or partners. In some federal member states, coordination remains weak or irregular, and national guidelines are not always uniformly implemented. Furthermore, while coordination forums offer structure, they are still limited by unequal power dynamics. Donors, as the primary financiers, often hold disproportionate influence in agenda setting and decision-making. The Ministry’s role is often reactive rather than directive, especially when capacity constraints or political tensions affect its operational autonomy. In this context, the sustainability of these mechanisms hinges not only on procedural consistency, but also on long-term investments in leadership development, data systems, and financial governance. Despite these challenges, recent shifts toward the localization of aid and the integration of vertical programs into the EPHS framework offer hope for improved synergy between aid and governance. Strengthening coordination platforms, embedding accountability structures, and reinforcing the Ministry’s regulatory role are critical steps toward transitioning from donor-dependence to sovereign health governance.

### Consequences of prolonged aid dependency

#### Systemic fragility and lack of sustainability

Prolonged reliance on external aid has entrenched systemic fragility in many post-conflict and developing countries, especially those with protracted emergencies such as Somalia. Rather than fostering institutional resilience, foreign assistance often delivered through parallel systems—undermines local ownership and capacity development. Health systems, in particular, have become overly dependent on donor-driven priorities and short-term projects, leading to limited investment in long-term health infrastructure and workforce development. As donors exit or reallocate funding, the collapse of essential services is common, highlighting the unsustainable nature of many aid-supported interventions [63]. In Somalia, the World Health Organization has emphasized the chronic underinvestment in core health system functions, with government spending on health among the lowest globally [18]. This has left the country vulnerable to recurring health crises, with weak surveillance, inadequate disease response, and low immunization coverage being persistent challenges [64–66].

#### Fragmentation and duplication of services

The influx of numerous international and non-governmental organizations, often operating in silos, has contributed to the fragmentation and duplication of services Somalia. With limited coordination mechanisms, actors frequently implement parallel programs in the same geographical regions, targeting overlapping beneficiary groups while neglecting others. This results in inefficiencies, inequitable access to services, and missed opportunities for synergy [67]. In Somalia, multiple assessments have found that humanitarian and development actors implement health, nutrition, and WASH programs with limited integration or alignment to national strategies [68]. The absence of a strong regulatory authority and the proliferation of vertical programs each with its own data systems, protocols, and reporting mechanisms has weakened the health information system, obscured accountability, and hindered the development of a unified health delivery platform [69, 70].

#### Financial dependency and policy misalignment

Aid dependency has fostered a fiscal environment where national priorities are often subordinated to donor preferences. Since a significant portion of public health financing in aid-dependent countries is off-budget, governments have minimal control over resource allocation or program design. This undermines national policy coherence, distorts budgeting processes, and complicates efforts to establish sustainable domestic revenue sources [71, 72]. In Somalia, donor funds account for over 80% of total health expenditure, with minimal domestic investment in preventive and promotive health services [21]. Policy misalignment is particularly evident in program planning, where donor cycles and conditions dictate implementation timelines, staffing patterns, and even intervention choices, often without adequate contextualization. As a result, health programs risk being misaligned with community needs and national health priorities, weakening the ability of governments to lead and coordinate sector-wide development [73, 74].

#### Political economy of aid

The political economy of aid reflects an imbalance in decision-making power, where donors—rather than recipient governments retain control over how resources are allocated and which priorities are addressed. This dynamic can undermine state legitimacy and accountability, especially when citizens perceive international actors as the primary service providers [75]. In fragile states like Somalia, where state institutions are still evolving, such dynamics have entrenched a parallel governance structure wherein international NGOs and UN agencies wield significant influence over policy and resource flows. The result is a weakened social contract between the state and its citizens, limiting the political incentive to invest in public goods [76]. Moreover, aid has at times reinforced elite capture, with powerful actors leveraging access to donor funds to maintain patronage networks or exclude opposition groups [77]. The risk of politicization of aid is further compounded by weak regulatory frameworks and limited civic engagement, making it difficult to ensure transparency, accountability, and equitable distribution of resources [21].

### Lessons from global/regional comparisons insights

Fragile and post-conflict countries often face similar dilemmas: rebuilding health systems with limited institutional capacity, high disease burden, political instability, and heavy reliance on donor-driven aid. Somalia’s experience mirrors that of other post-conflict settings such as South Sudan, Liberia, Afghanistan, and Sierra Leone, where aid played a central role in reviving the health sector. Comparative analysis of these cases offers valuable insights into what has worked, what has not, and the pathways that could lead Somalia towards more sustainable health governance (Table 4).

**Table 4:**
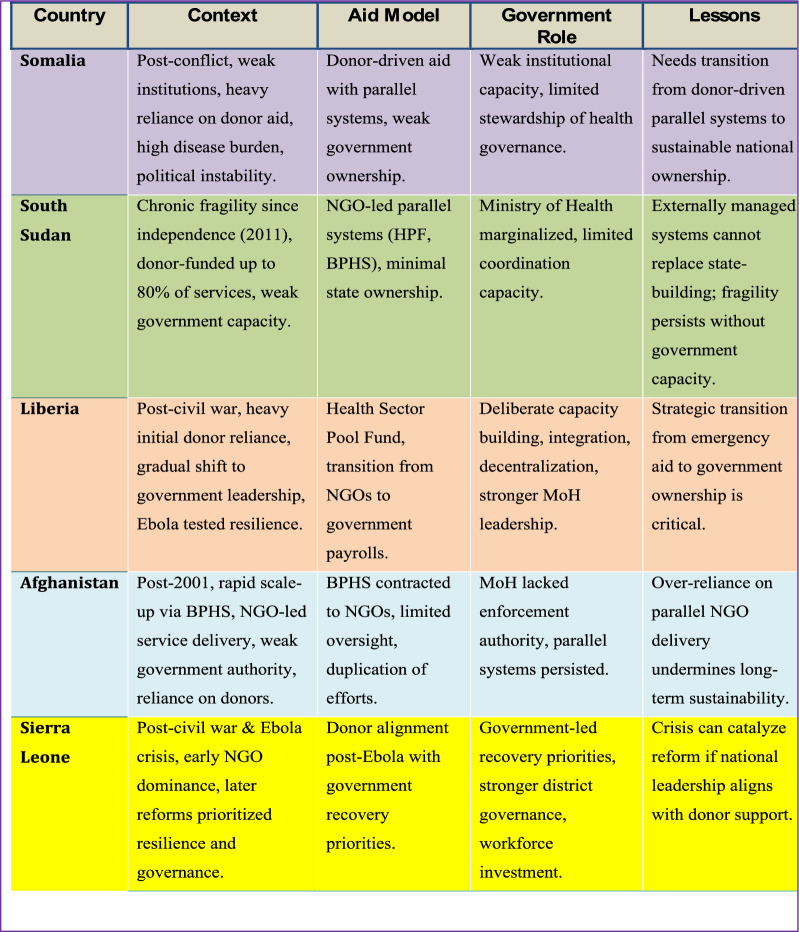
Comparative insights: health system recovery in fragile states

#### South Sudan: aid amidst chronic fragility

South Sudan, like Somalia, has remained in a prolonged state of fragility with limited government capacity. Following independence in 2011, the country became heavily reliant on humanitarian aid, with donors funding up to 80% of its health services through the Health Pooled Fund (HPF) and Basic Package of Health Services (BPHS) [78–80]. While these arrangements temporarily expanded access to primary care in underserved areas, they were implemented through parallel systems managed by NGOs, limiting government ownership and weakening the role of the Ministry of Health [81]. Coordination challenges and lack of long-term capacity-building investments have left South Sudan vulnerable to health system collapse whenever funding fluctuates or insecurity escalates [82]. This highlights the limitations of externally managed health architectures without concurrent state-building efforts.

#### Liberia: a gradual shift to government leadership

Liberia provides a contrasting trajectory. Following its civil war, Liberia also relied heavily on external funding and NGO-led delivery of services. However, starting in the mid-2000s, the country made deliberate efforts to strengthen public sector capacity and reclaim leadership in health governance [83]. The government introduced the National Health Policy and Plan (2011–2021) with a strong focus on integration, decentralization, and system-wide reforms [84]. Donor alignment improved through mechanisms like the Health Sector Pool Fund and support for civil service reform, which transitioned health workers from NGO to government payrolls [85]. Although the Ebola outbreak in 2014 exposed lingering weaknesses, Liberia’s post-Ebola strategy further accelerated government stewardship, improved disease surveillance, and expanded community health worker programs [86, 87]. The lesson here is the importance of strategic transition planning from emergency aid to long-term institutional capacity.

#### Afghanistan: parallel systems and lost opportunities

Afghanistan, despite initial success in scaling up service delivery through the Basic Package of Health Services (BPHS) model funded by donors such as USAID, World Bank, and the EU, suffered from structural limitations due to persistent use of parallel systems [88, 89]. The government contracted NGOs to deliver services, but often lacked authority to oversee implementation or enforce standards [90, 91]. This led to inefficiencies, duplication, and an underdeveloped public sector that struggled to sustain services when donor support declined especially during periods of political transition [92, 93]. Afghanistan’s experience illustrates the pitfalls of over-reliance on externally managed delivery models without a clear strategy to transfer responsibilities to national systems.

#### Sierra Leone: building resilience after crisis

Sierra Leone's health system was severely weakened by civil war and later devastated by the Ebola crisis. Initial recovery efforts were dominated by humanitarian actors. However, post-Ebola reforms brought stronger emphasis on resilience and governance. The introduction of the Presidential Recovery Priorities and National Health Sector Recovery Plan (2015–2020) focused on rebuilding essential services while investing in health workforce, infrastructure, and district-level governance [94, 95]. Although implementation was uneven, Sierra Leone benefited from donor alignment with government priorities and increased domestic health financing [96]. The experience suggests that crisis-driven reform windows can catalyze deeper structural improvements if national leadership and donor coordination are aligned.

#### Strategic implications for Somalia’s health-system recovery

The comparative experiences of post-crisis health system recovery provide key lessons for Somalia. Government ownership and leadership in health policy, financing, and workforce management are crucial for ensuring sustainability. While external aid can stabilize health services during crises, it must be strategically aligned with institutional capacity-building goals to avoid dependency. Strengthening national governance structures is essential for ensuring that aid supports long-term development, rather than short-term relief.

Effective donor coordination and integration into national systems are critical for reducing fragmentation and improving operational efficiency. Liberia and Sierra Leone illustrate how pooled funds and joint planning frameworks can foster greater alignment, accountability, and efficiency among international partners. These approaches contribute to a more cohesive and sustainable health system, facilitating smoother transitions between humanitarian aid and long-term health sector strengthening.

Finally, Somalia must prioritize transition planning from humanitarian relief to sustainable development models. As seen in South Sudan and Afghanistan, reliance on parallel systems without a structured exit strategy or capacity-building roadmap undermines long-term resilience. Somalia should leverage its ongoing state-building process to define a clear national vision for health system recovery, focusing on accountability, harmonizing partner efforts with the Ministry of Health’s strategic priorities, and gradually shifting toward domestic resource mobilization. Without this shift, the country risks perpetuating a cycle of fragility that has hindered progress in many post-conflict settings.

Somalia’s health sector transition from relief to sustainable development necessitates developmental models that prioritize system-building. These models focus on strengthening institutional capacity, integrating health systems, and making long-term investments in infrastructure, workforce, and governance. Key approaches include the Sector-Wide Approach (SWAp), which aligns donor aid with national priorities under government leadership, and Health Systems Strengthening (HSS), targeting the six WHO building blocks: service delivery, workforce, information systems, medicines, financing, and leadership. Community health strategies and public-private partnerships are also crucial for expanding reach and resilience. This coordinated shift from vertical aid to developmental models fosters ownership, accountability, and sustainable health outcomes.

### Pathways toward sustainability Somalia’s health system

Achieving sustainability in health systems post-conflict, especially in a context like Somalia, requires a concerted shift from donor-driven interventions to locally owned, government-led strategies. The long-standing reliance on external aid in Somalia has created both opportunities and challenges in the pursuit of a self-sustaining health system. To ensure that Somalia’s health system can withstand future shocks without returning to a state of dependency, several pathways must be pursued.

#### Strengthening national governance and institutional capacity

One of the central tenets of transitioning toward sustainability is strengthening national governance. The Somali government, particularly the Federal Ministry of Health (FMoH), must be at the heart of health policy planning and decision-making. Donor aid should be integrated into the broader government-led frameworks such as the Health Sector Strategic Plan (HSSP III) and Essential Package of Health Services (EPHS), which should be systematically implemented and adequately resourced. The government must enhance its ability to oversee and regulate health service delivery, moving away from parallel systems managed by NGOs and international agencies. Building the capacity of the FMoH to effectively lead the health sector will require sustained investment in human resources, infrastructure, and technical expertise. This includes training health workers, improving data management systems, and enhancing governance structures at the federal and regional levels. By ensuring that the government takes an active role in the design, implementation, and monitoring of health programs, the risk of fragmentation and inefficiencies will decrease, and local ownership will increase.

#### Transitioning from humanitarian aid to long term

Development The shift from humanitarian aid to long-term development is critical for sustainability. In many post-conflict countries, aid is often framed as short-term emergency relief, which, while essential, does not adequately address the long-term needs of the population. In Somalia, humanitarian aid has frequently been provided through vertical programs that target specific diseases, which are important but do not foster long-term health system strengthening. A more effective approach would involve integrating emergency programs into a broader development strategy that aligns with national health priorities. This could be achieved through the development of pooled funding mechanisms, where donors contribute to a single fund that is managed by the government or a designated authority. This would promote better coordination, reduce inefficiencies, and ensure that donor funding is aligned with the country’s long-term health goals, such as the strengthening of primary health care, non-communicable disease control, and mental health services.

#### Promoting domestic resource mobilization

While international aid has played a pivotal role in funding Somalia’s health sector, a major hurdle for long-term sustainability is the country’s limited ability to finance health services domestically. Somalia must prioritize strategies that will allow it to mobilize domestic resources for health. This could involve improving tax revenue collection, establishing dedicated health funds, and exploring public-private partnerships to finance critical health infrastructure. Additionally, international donors can play a role in facilitating the establishment of financial sustainability models. For example, encouraging governments to contribute a greater share of the national budget to health and advocating for domestic revenue generation would reduce dependency and allow for more flexibility in meeting the country’s evolving health needs.

#### Enhancing coordination among donors and partners

The multiplicity of international donors and NGOs in Somalia has created a fragmented health system. To improve sustainability, there must be a focus on improving donor coordination. The Health Sector Coordination Forums (HSCFs), which include representatives from the government, UN agencies, NGOs, and donors, play a critical role in ensuring that all partners are aligned with national health priorities. These forums can serve as platforms for joint planning, resource mapping, and decision-making. Strengthening these coordination mechanisms will help prevent duplication of efforts and allow for a more holistic approach to health service delivery. It is crucial that all stakeholders, including donors, align their funding and programs with Somalia’s long-term health objectives. Through increased coordination, it will be possible to create synergies between different sectors and reduce gaps in service provision.

#### Building community resilience and engagement

A crucial aspect of sustainability is ensuring that communities are actively involved in health service delivery and decision-making. Empowering communities to take ownership of health initiatives will not only improve the utilization of services but also foster a sense of responsibility and accountability. Community health workers, who are often on the frontlines of service delivery in rural areas, must be adequately trained, equipped, and integrated into the broader health system. Moreover, community-based health insurance models could provide financial protection against out-of-pocket expenditures for health services, which would reduce reliance on external funding. These models could be piloted in regions with strong community cohesion, providing both financial sustainability and better access to essential health services.

#### Transitioning health workforce from NGO to government payroll

A major challenge in Somalia’s health sector is the reliance on NGO-led delivery models that often do not integrate national health workers into the system. Over time, it will be crucial for Somalia to transition its health workforce from NGO payrolls to the government’s health system. This would involve increasing government recruitment and ensuring the integration of health workers into the public sector, providing them with the necessary training, financial incentives, and career development opportunities. A well-trained and adequately compensated workforce will contribute to the long-term sustainability of the health system, as it will ensure the continuity of services even when external support decreases. This transition should be aligned with efforts to improve human resource management within the Ministry of Health and local health authorities.

## Discussion

The prolonged reliance on multisectoral aid in Somalia’s post-conflict era has undeniably shaped the trajectory of its health system and governance structures. While international assistance has been instrumental in addressing immediate health crises and rebuilding basic service delivery, the findings of this review highlight critical tensions between short-term gains and long-term sustainability. The Somali experience mirrors broader patterns observed in fragile and post-conflict states, where aid dependency often perpetuates systemic fragility, undermines local ownership, and complicates the transition to self-sustaining governance [97].

This discussion synthesizes key themes, draws comparative insights, and proposes actionable pathways forward.

International aid has yielded measurable improvements in Somalia’s health outcomes, particularly in maternal and child health (MCH), immunization, and disease control [98]. Programs like the Expanded Programme on Immunization (EPI) and the Global Fund’s malaria and TB initiatives have saved lives and mitigated epidemics [42]. However, these successes are tempered by the dominance of vertical, donor-driven programs that operate in silos, bypassing national systems [99]. The lack of integration with Somalia’s Essential Package of Health Services (EPHS) and the proliferation of parallel structures such as NGO-managed supply chains and health information systems have exacerbated fragmentation [100]. This fragmentation not only duplicates efforts but also weakens the Federal Ministry of Health’s (FMoH) capacity to enforce standards or coordinate sector-wide priorities [18].

The tension between weak state capacity and donor influence is a recurring theme in Somalia’s post-conflict recovery. While FMoH has developed policy frameworks like the Health Sector Strategic Plan (HSSP III), its implementation remains constrained by limited technical expertise, funding gaps, and the outsized role of external actors in decision-making [18]. Comparative cases, such as Afghanistan and South Sudan, illustrate how prolonged reliance on parallel systems can erode government legitimacy and institutionalize dependency, hindering long-term development and resilience [101]. In Somalia, donor priorities often overshadow national needs, particularly in underfunded areas like non-communicable diseases (NCDs) and mental health [102, 103]. The Health Sector Coordination Forums (HSCFs), though a step toward alignment, remain hampered by power asymmetries, with donors wielding disproportionate influence over agendas [59].

The experiences of Liberia and Sierra Leone offer valuable contrasts. Liberia’s post-war shift toward government-led health governance, including the integration of NGO workers into public payrolls, demonstrates the importance of deliberate capacity-building [104]. Similarly, Sierra Leone’s post-Ebola reforms prioritized resilience through domestic financing and district-level governance [105, 106]. These cases underscore that true sustainability depends on two fundamental pillars: aligning donor support with national capacity development and shifting from short-term humanitarian relief to long-term health system strengthening [105, 107]. Somalia’s federal structure adds complexity, but lessons from these countries suggest that pooled funding mechanisms and phased transitions such as gradually shifting NGO-managed services to government oversight could mitigate fragmentation [18].

Somalia’s health gains remain fragile—despite strong donor-supported outcomes, funding cuts expose risks of disease and malnutrition. Lasting progress depends on strengthening institutions, building local ownership, and prioritizing primary care for sustainable resilience. Leveraging existing donor supports to build national systems such as integrating parallel data platforms and harmonizing procurement can gradually shift operational control to the government. Promoting community-based care, expanding health education, and deploying digital health tools can improve outreach and efficiency. Moreover, aligning aid with national priorities through sector-wide approaches and improving public financial management will enhance transparency and foster donor confidence, setting the stage for increased domestic and pooled funding in the long term.

Investing in Somalia’s national epidemic preparedness and response capacity is essential due to its fragile political and geographical context and high vulnerability to outbreaks. Key strategies include strengthening disease surveillance, enhancing laboratory networks, training rapid-response teams, and improving coordination among federal, state, and community levels. Sustained donor alignment and government ownership are also crucial for bolstering resilience against infectious threats and reinforcing overall health system development.

To break the cycle of dependency, Somalia must pursue multifaceted strategies:

***Strengthening government leadership.*** The FMoH must transition from a passive coordinator to an active steward of health services. This requires investing in technical capacity, data systems, and regulatory frameworks to oversee integrated primary health care (PHC) [21].

***Domestic resource mobilization.*** With over 80% of health funding externally sourced, Somalia must explore innovative financing, such as health taxes or public–private partnerships, to reduce reliance on volatile aid flows [21].

***Donor harmonization.*** Coordination platforms like HSCFs should be empowered to enforce alignment with national plans, reducing duplication and improving accountability [108].

***Community engagement.*** Community health workers and localized insurance models can enhance resilience and ownership, as seen in Rwanda’s mutuelle system [109, 110].

In Somalia, civil-society groups and traditional leaders can help strengthen the health system by co-managing Sharia-compliant insurance funds, overseeing community health workers, and using public scorecards to link household contributions to health outcomes—promoting accountability, financial resilience, and community ownership [111, 112].

## Recommendation

### Innovative health financing stack (SINTAX)

Combine earmarked excises (tobacco, sugary drinks), a tiny mobile-money micro-levy, a diaspora “Health Bond”, and structured Zakat/Waqf channels into a ring-fenced Primary Health Care (PHC) fund; publish semi-annual revenue/outlay scorecards and legally lock proceeds to EPHS priorities and financial protection.

### Government-led health compact and SWAp

Sign a time-bound Health Compact that makes EPHS/HSSP the single plan, budget, and results matrix (SWAp): all partners align to one M&E dashboard, join biannual joint sector reviews, and use common costing/indicators—whether they pool funds or “shadow-align”—with corrective actions tied to next-period disbursements.

### Fiscal federalism with formula-based grants

Institute transparent, rules-based health grants from FGS to FMS using a formula (population, poverty, remoteness, need) plus a performance top-up for equity and quality; publish allocations and execution quarterly and include an equalization component for hard-to-reach districts. Strategic Purchasing and Provider Payment Reform Create a purchaser function to contract public, private, and faith-based providers on EPHS outputs: capitation for PHC, case-based payments for priority services, and quality bonuses; enable e-claims, independent verification, and patient feedback loops to drive value and reduce fragmentation.

### Public–private partnership (PPP) framework

Adopt a light, standardized PPP policy to crowd in private capital and capability for diagnostics, oxygen, maintenance, dialysis, and telehealth; use service-level agreements with clear tariffs, uptime, and quality KPIs, and tender competitively to cap unit costs and expand access.

### Community financing and protection (CBHI/Takaful)

Pilot Sharia-compliant micro-takaful/CBHI in stable districts with mobile payments, basic benefits mapped to EPHS, and subsidies for the poorest via an equity fund; seat community oversight boards and link enrollment to facility quality scorecards to build trust and sustained uptake.

### Digital and data governance “one stack”

Mandate an interoperable digital stack: DHIS2 as the national warehouse, unified data dictionary/APIs, integrated IDSR/EMR/PHEOC feeds, and unique health IDs; require all partners to push routine data monthly, auto-generate public dashboards, and use data for performance payments and targeted supervision.

### Unified supply chain and medicines regulation

Collapse parallel supply chains into a single national quantification and framework contracts, backed by an electronic LMIS with real-time stock visibility to the last mile; strengthen the regulator for market authorization, GMP/GDP inspections, and pharmacovigilance to ensure quality and cut stockouts/leakage.

### Workforce transition and HRH pipeline

Map NGO-funded posts, harmonize pay scales, and phase front-line absorption onto the government payroll where fiscal space allows; scale accredited CHWs and mid-level cadres with task-shifting (e.g., nurse-led NCD/mhGAP), deploy an HRIS for postings/payroll/licensure, and fund rural hardship/retention packages.

### Emergency preparedness and risk financing

Operationalize a PHEOC with district rapid-response teams, sentinel labs, and simulated surge plans; create a contingency budget line with pre-approved emergency procurement and explore parametric drought/outbreak insurance; align humanitarian actors to an EPHS “bridge package” to smooth the HDP nexus during shocks.

## Limitations

This narrative review is subject to several limitations. First, while the study draws on a broad range of peer-reviewed literature and grey sources, it is inherently limited by the availability and quality of secondary data. Much of the documentation on aid effectiveness and health governance in Somalia is programmatic or donor-driven, which may carry implicit biases or lack independent verification. Second, due to the fragmented nature of the Somali health system and limited data centralization, there may be gaps in capturing the full extent of regional variations or subnational dynamics, especially in federal member states where aid delivery models differ. Third, the study focuses predominantly on health system dynamics and may underrepresent intersections with broader governance sectors such as justice, education, and economic development, which are equally relevant in understanding sustainability. Finally, while comparisons with other fragile states provide valuable insights, contextual differences may limit the generalizability of these lessons to Somalia's unique sociopolitical landscape.

## Conclusion

Somalia’s post-conflict health recovery has been deeply shaped by international aid filling critical service gaps, addressing disease outbreaks, and supporting key maternal and child health interventions. Yet, the prolonged reliance on multisectoral, donor-driven programs has reinforced systemic fragility, sidelined national leadership, and inhibited the development of a cohesive and sustainable health system. This review reveals that despite improvements in health indicators, fragmentation, vertical programming, and parallel structures persist, undermining Somalia’s ability to transition from emergency response to long-term health governance and future depends on self-reliance through stronger systems and primary care.

To break free from aid dependency, Somalia must focus on strengthening government leadership, mobilizing domestic resources, and ensuring donor efforts align with national strategies. The experiences of other post-conflict nations such as Liberia’s integration of NGO health workers or Sierra Leone’s post-Ebola reforms illustrate that sustainability is not only possible, but imperative. Aligning external support with institutional capacity-building, enhancing coordination platforms like the Health Sector Coordination Forums, and fostering community-driven service delivery models are crucial steps. Ultimately, building a resilient and self-reliant health system will require a phased, deliberate, and locally led transition from aid to autonomy—anchored in policy coherence, fiscal responsibility, and inclusive governance.

## Data Availability

As this is a narrative review, no primary datasets were generated. However, the authors remain available to share supporting materials or references upon reasonable request to facilitate transparency and replication.

## Declarations

### Descriptions for experiments

No experiments involving human or animal subjects were conducted. This study is based on a narrative review of published literature and publicly available data sources.

## References

[1] Ssengooba F, Namakula J, Kawooya V, Fustukian S. Sub-national assessment of aid effectiveness: a case study of post-conflict districts in Uganda. Glob Health. 2017. 10.1186/s12992-017-0251-7. 10.1186/s12992-017-0251-7 PMC5470258 28610578

[2] Bennett S, Glandon D, Rasanathan K. Governing multisectoral action for health in low-income and middle-income countries: unpacking the problem and rising to the challenge. BMJ Glob Health. 2018;3(Suppl 4):880. 10.1136/bmjgh-2018-000880 PMC6195144 30364411

[3] Sutarsa IN, Campbell L, Ariawan IMD, Kasim R, Marten R, Rajan D, et al. Multisectoral interventions and health system performance: a systematic review. Bull World Health Organ. 2024;102(7):521. 38933474 10.2471/BLT.23.291246 PMC11197648

[4] Martineau T, McPake B, Theobald S, Raven J, Ensor T, Fustukian S, et al. Leaving no one behind: lessons on rebuilding health systems in conflict- and crisis-affected states. BMJ Glob Health. 2017;2(2):e000327. 29082000 10.1136/bmjgh-2017-000327 PMC5656126

[5] Swenson G, Kniess J. International assistance after conflict: health, transitional justice and opportunity costs. Third World Q. 2021;42(8):1696–714. 10.1080/01436597.2021.1928489.

[6] Viphonephom P, Kounnavong S, Reinharz D. Decentralization and immunization program in a single-party state: the case of the Lao People’s Democratic Republic. Trop Med Health. 2024. 10.1186/s41182-024-00601-8. 38715093 10.1186/s41182-024-00601-8 PMC11075326

[7] Waga AA. Navigating chaos: IGAD’s efforts Amidst Somalia’s Governance crisis. Heliyon. 2024;10(18):e37941. 39315161 10.1016/j.heliyon.2024.e37941 PMC11417544

[8] Dynamics in the Horn of Africa region and the interests of the Republic of Turkey | National Institute for Strategic Studies. [cited 2025 Apr 7]. Available from: https://niss.gov.ua/en/doslidzhennya/mizhnarodni-vidnosini/dynamics-horn-africa-region-and-interests-republic-turkey.

[9] Somalia: Colonialism to Independence to Dictatorship, 1840–1976–the Enough Project. [cited 2025 Apr 7]. Available from: https://enoughproject.org/blog/somalia-colonialism-independence-dictatorship-1840-1976.

[10] Menkhaus K. State collapse in Somalia: second thoughts. Rev Afr Polit Econ. 2003;30(97):405. 10.1080/03056244.2003.9659774.

[11] Somali Civil War–the Organization for World Peace. [cited 2025 Apr 6]. Available from: https://theowp.org/crisis_index/somali-civil-war/.

[12] Mourad KA. Post-conflict development, reviewing the water sector in Somalia. Environ Dev Sustain. 2023;25(2):1326–50. 10.1007/s10668-021-02096-3. 35002479 10.1007/s10668-021-02096-3 PMC8721627

[13] Ahmed N, Mohamud Ahmed N. Somalia’s struggle to integrate traditional and modern Somalia’s struggle to integrate traditional and modern governance: the 4.5 formula and 2012 provisional constitution governance: the 4.5 formula and 2012 provisional constitution. [cited 2025 Apr 7]; Available from: https://fount.aucegypt.edu/etds.

[14] (PDF) The Effect of International Intervention on Political Stability in Mogadishu, Somalia. [cited 2025 Apr 7]. Available from: https://www.researchgate.net/publication/380493876_The_Effect_of_International_Intervention_on_Political_Stability_in_Mogadishu_Somalia.

[15] Farah I. Somalia: thirty years after. Development (Rome). 2021 Jun 1 [cited 2025 Apr 7];64 (1–2):107. Available from: https://pmc.ncbi.nlm.nih.gov/articles/PMC8050483/.

[16] Annual Budget | Ministry of Finance-Somalia. [cited 2025 Apr 19]. Available from: https://mof.gov.so/publications/annual-budget.

[17] CONSOLIDATED ANNUAL FINANCIAL REPORT of the Administrative Agent UN Multi-Partner Trust Fund Office United Nations Development Programme GATEWAY: https://mptf.undp.org. 2025 [cited 2025 Sep 18]; Available from: https://mptf.undp.org.

[18] Said AS, Kicha DI. Implementing health system and the new federalism in Somalia: challenges and opportunities. Front Public Health. 2024;12:1205327. 38362207 10.3389/fpubh.2024.1205327 PMC10867962

[19] Ahmed Z, Ataullahjan A, Gaffey MF, Osman M, Umutoni C, Bhutta ZA, et al. Understanding the factors affecting the humanitarian health and nutrition response for women and children in Somalia since 2000: a case study. Confl Health. 2020;14(1):35. 32514300 10.1186/s13031-019-0241-x PMC7254682

[20] Mohamed IA. The Impact of International aid on state building Stability and Development: Case study of Somalia. [cited 2025 Apr 7]; Available from: www.allmultidisciplinaryjournal.com.

[21] Ahmed AY, Nor FA, Ahmed MY, Osman MM, Ahmed AY, Nor FA. Universal health coverage in Somalia: charting the path to equitable healthcare financing and governance. Health. 2023;15(11):1298–317.

[22] The Somali Investment Case for Reproductive.

[23] Somalia Monthly Humanitarian Update, April 2025-Somalia | ReliefWeb. [cited 2025 Sep 18]. Available from: https://reliefweb.int/report/somalia/somalia-monthly-humanitarian-update-april-2025.

[24] Disease spreads in Somalia as funding cuts leave 300,000 without safe water-Somalia | ReliefWeb. [cited 2025 Sep 18]. Available from: https://reliefweb.int/report/somalia/disease-spreads-somalia-funding-cuts-leave-300000-without-safe-water.

[25] Somalia: Monthly Humanitarian Update, July 2025 | OCHA. [cited 2025 Sep 18]. Available from: https://www.unocha.org/publications/report/somalia/somalia-monthly-humanitarian-update-july-2025.

[26] Dahir A, Sheikh Ali AY. Federalism in post-conflict Somalia: a critical review of its reception and governance challenges. Reg Fed Stud. 2024;34(1):87–106. 10.1080/13597566.2021.1998005.

[27] Salad AM, Malik SMMR, Ndithia JM, Noor Z, Madeo M, Ibrahim M. Prevalence of mental disorders and psychological trauma among conflict- affected population in Somalia: a cross-sectional study. Front Public Health. 2023;11:1219992. 37829096 10.3389/fpubh.2023.1219992 PMC10565346

[28] Hussein SA, Osman MM, Abdulle YS, Hussein AA, Md AAT, Nur AM, et al. Knowledge, attitude, and practice of HIV/AIDS transmission and prevention among barbing and beauty salon operators in Mogadishu, Somalia, 2024. BMC Public Health. 2025;25(1):696. 39979892 10.1186/s12889-025-21945-8 PMC11841181

[29] Border management–immigration and citizenship agency. [cited 2024 Nov 4]. Available from: https://immigration.gov.so/border-management/.

[30] Haddaway NR, Page MJ, Pritchard CC, McGuinness LA. PRISMA2020: an R package and Shiny app for producing PRISMA 2020-compliant flow diagrams, with interactivity for optimised digital transparency and Open Synthesis. Campbell Syst Rev. 2022;18(2):e1230. 36911350 10.1002/cl2.1230 PMC8958186

[31] UNOSOM | United Nations Peacekeeping Mission in Somalia | Britannica. [cited 2025 Apr 8]. Available from: https://www.britannica.com/topic/UNOSOM.

[32] Hussein MA. The effects of aid in Somalia: unintended consequences and lessons learned. J Econ Coop Dev. 2024;45:209–32.

[33] Somalia: a health system in crisis-Somalia | ReliefWeb. [cited 2025 Apr 8]. Available from: https://reliefweb.int/report/somalia/somalia-health-system-crisis.

[34] Warsame AA. Human Capital Development Strategy. 2020.

[35] Maalim AM, Zachariah R, Khogali M, Van griensven J, Van den bergh R, Tayler-Smith K, et al. Supporting ‘medicine at a distance’ for delivery of hospital services in war-torn Somalia: how well are we doing? Int Health. 2014;6 (1):70–3. 10.1093/inthealth/iht035. 10.1093/inthealth/iht035 24431137

[36] Overcoming fragility in Somalia to build a strong primary health care system. [cited 2025 Apr 8]. Available from: https://www.who.int/news-room/feature-stories/detail/somalia.

[37] Jama MA, Majdzadeh R, Reynolds T, Nur IM, Ismail AA, Mohamud NA, et al. Revising the essential package of health services through stakeholder alignment, Somalia. Bull World Health Organ. 2023;101(11):738. 37961055 10.2471/BLT.23.289733 PMC10630727

[38] Analysis of aid flow data Aid Flows in Somalia. 2017.

[39] ICRC Health Response in Somalia. [cited 2025 Apr 8]. Available from: https://www.icrc.org/en/document/icrc-health-response-somalia.

[40] Hayir TMM, Magan MA, Mohamed LM, Mohamud MA, Muse AA. Barriers for full immunization coverage among under 5 years children in Mogadishu, Somalia. J Family Med Prim Care. 2020;9(6):2664. 32984104 10.4103/jfmpc.jfmpc_119_20 PMC7491846

[41] Bile AS, Ali-Salad MA, Mahmoud AJ, Singh NS, Abdelmagid N, Sabahelzain MM, et al. Assessing vaccination delivery strategies for zero-dose and under-immunized children in the fragile context of Somalia. Vaccines. 2024;12(2):154. 38400137 10.3390/vaccines12020154 PMC10892412

[42] Somalia immunization programme: catching up after conflict and COVID-19 in a fragile setting CASE STUDY. 2023.

[43] Somali Private Sector Partnerships in Health | Market Systems Assessment ii.

[44] Health Sector Resource Mapping and Expenditure Tracking Report. 2021.

[45] WHO EMRO | Integrated Disease Surveillance and Response system: a game changer in Somalia | News | Somalia site. [cited 2025 Apr 8]. Available from: https://www.emro.who.int/somalia/news/integrated-disease-surveillance-and-response-system-a-game-changer-in-somalia.html.

[46] Ssendagire S, Karanja MJ, Abdi A, Lubogo M, Azad Al A, Mzava K, et al. Progress and experiences of implementing an integrated disease surveillance and response system in Somalia; 2016–2023. Front Public Health. 2023;11:1204165. 37780418 10.3389/fpubh.2023.1204165 PMC10539911

[47] Somalia: Disease surveillance and response strategy amid fragility | PreventionWeb. [cited 2025 Apr 8]. Available from: https://www.preventionweb.net/news/somalia-pioneers-implementation-integrated-disease-surveillance-and-response-strategy-fragile.

[48] WHO EMRO | World Malaria Day 2023–zeroing in on malaria | News | Somalia site. [cited 2025 Apr 8]. Available from: https://www.emro.who.int/somalia/news/world-malaria-day-2023-zeroing-in-on-malaria.html.

[49] The National Strategic Plan for Tuberculosis Control 2024–2026 National Leprosy and Tuberculosis Control Program Somalia ministry of health Somalia ministry of health Somalia Ministry Of Health. 2023.

[50] About Health | ReliefWeb Response. [cited 2025 Apr 8]. Available from: https://response.reliefweb.int/somalia/health/about-health.

[51] HPC-Project Module: View Project. [cited 2025 Apr 8]. Available from: https://projects.hpc.tools/project/151598/view.

[52] The Multi-sectoral Minimum Response Package in Somalia | ENN. [cited 2025 Apr 8]. Available from: https://www.ennonline.net/resource/nutrition-exchange/multi-sectoral-minimum-response-package-somalia.

[53] Investment Case for the Somalia Health Sector 2022–2027.

[54] Mothupi M, Ahmed MA, Mohamud AM, Dalmar A, Jimale MAO, Abdullahi H, et al. Maternal and newborn health prioritization in post-transition Somalia: analysis of key stakeholder perspectives at the federal level. SSM Health Syst. 2025;4:100072.

[55] Spicer N, Agyepong I, Ottersen T, Jahn A, Ooms G. “It’s far too complicated”: why fragmentation persists in global health. Glob Health. 2020. 10.1186/s12992-020-00592-1. 10.1186/s12992-020-00592-1 PMC7344046 32646471

[56] Private Sector Partnerships in Health (PSPH) (Somalia & Somaliland). [cited 2025 Apr 8]; Available from: https://www.eda.admin.ch/countries/kenia/en/home/international-cooperation/themes.html.

[57] Ministry of Health and Human Services Federal Republic of Somalia Officially Announced on November-9th 2023 Event: Launching of the Alignment Partnership Principles in Somalia’s Health Sector at Mercure Hotel. Upper Hill Nairobi, Kenya Joint Statement of Partnership Principles Supporting Alignment and Harmonization in Somalia’s Health Sector.

[58] Hidig SM. Assessing healthcare challenges in Somalia: a 2024 perspective. Ankara City Hospital Med J. 2024;3(1):47–8.

[59] Overcoming fragility in Somalia to build a strong primary health care system. [cited 2025 Jan 1]. Available from: https://www.who.int/news-room/feature-stories/detail/somalia.

[60] Kahow MH, Halane SA, Ali A, Shah R. ‘Health camp’ model: a unique approach for child vaccination in non-state armed actor controlled, inaccessible geographies in Somalia. Glob Health Action. 2024. 10.1080/16549716.2024.2391598. 39175410 10.1080/16549716.2024.2391598 PMC11378116

[61] Ministry of Health and Human Services Federal Government of Somalia. 2021.

[62] Alignment - UHC2030. [cited 2025 Sep 14]. Available from: https://www.uhc2030.org/alignment/.

[63] Aid effectiveness in fragile states: How bad is it and how can it improve?. [cited 2025 Apr 8]. Available from: https://www.brookings.edu/articles/aid-effectiveness-in-fragile-states/.

[64] Somalia: The deadly consequences of obstacles to health care | Doctors without Borders - USA. [cited 2025 Apr 8]. Available from: https://www.doctorswithoutborders.org/latest/somalia-deadly-consequences-obstacles-health-care.

[65] Policy-Brief-on-The-Health-Economic-and-Social-Impact-of-Covid-19-in-Somalia.

[66] strengthening-public-health-systems-case-study-august-1_2023.

[67] Warsame A, Frison S, Checchi F. Drought, armed conflict and population mortality in Somalia, 2014–2018: a statistical analysis. PLoS Glob Public Health. 2023;3(4):e0001136. 10.1371/journal.pgph.0001136. 37043439 10.1371/journal.pgph.0001136 PMC10096495

[68] Inter-agency humanitarian evaluation of the response to the humanitarian crisis in Somalia evaluation report Management, Funding, and Implementation of the Evaluation. 2025.

[69] (PDF) The challenges facing the healthcare system in Somalia: a review and the way forward. [cited 2025 Apr 8]. Available from: https://www.researchgate.net/publication/378108504_The_challenges_facing_the_healthcare_system_in_Somalia_A_review_and_The_Way_Forward.

[70] Comprehensive assessment of Somalia’s health information system 2022.

[71] Somalia: pathways to Economic and Institutional Reforms, Peace and Reconciliation, Environmental Restitution, and Sustainable development. 2022.

[72] Brown GW, Rhodes N, Tacheva B, Loewenson R, Shahid M, Poitier F. Challenges in international health financing and implications for the new pandemic fund. Glob Health. 2023. 10.1186/s12992-023-00999-6. 10.1186/s12992-023-00999-6 PMC10696881 38053177

[73] Somalia: Building sustainable private sector healthcare financing and service networks-DTGlobal. [cited 2025 Apr 8]. Available from: https://dt-global.com/projects/psph/.

[74] Investing in Health to Anchor Growth.

[75] Stiftung B. BTI 2024 Country Report Somalia. [cited 2025 Apr 8]; Available from: https://www.bti-project.org.

[76] Somalia’s survival depends on fundamental governance reform - Africa at LSE. [cited 2025 Apr 8]. Available from: https://blogs.lse.ac.uk/africaatlse/2024/08/30/somalias-survival-depends-on-fundamental-governance-reform/.

[77] Jaspars S, Majid N, Adan GM. Somalia’s evolving political market place: from famine and humanitarian crisis to permanent precarity. J Mod Afr Stud. 2023;61(3):343–66.

[78] Majak JCD, Dhieu BD. Public Health Leadership in Fragile States: Lessons from South Sudan’s Health System. Int J Res Sci Innov. 2025;XII (XV):398–409.

[79] Widdig H, Tromp N, Lutwama GW, Jacobs E. 123:poster The political economy of priority-setting for health in South Sudan: a case study of the health pooled fund. 2022;A16.3-A17. 10.1186/s12939-022-01665-w PMC9108706 35578242

[80] Obels I, Coleman HLS, Straetemans M, van Gurp M, Lutwama GW, Jacobs E. Determinants of health seeking behaviour in South Sudan: a cross-sectional household survey. BMC Public Health. 2024;25(1):1–16. 10.1186/s12889-024-19798-8. 10.1186/s12889-024-19798-8 PMC11702148 39762807

[81] Malel ZJ, Lueth GD, Ayuel MM, Singba ND. Quality of care in South Sudan and its associated factors, a facility-based cross-sectional study in public health facilities in Yambio County, Western Equatorial State. BMC Health Serv Res. 2024. 10.1186/s12913-024-11728-z. 39533304 10.1186/s12913-024-11728-z PMC11559149

[82] South Sudan: Investing in critical health infrastructure for improved health delivery | WHO | Regional Office for Africa. [cited 2025 Apr 8]. Available from: https://www.afro.who.int/countries/south-sudan/news/south-sudan-investing-critical-health-infrastructure-improved-health-delivery.

[83] An analysis of Liberia’s post-civil war reconstruction efforts, the progress made, and the challenges that remain - FrontPageAfrica. [cited 2025 Apr 8]. Available from: https://frontpageafricaonline.com/opinion/commentary/an-analysis-of-liberias-post-civil-war-reconstruction-efforts-the-progress-made-and-the-challenges-that-remain/.

[84] Ministry of Health. [cited 2025 Apr 8]; Available from: https://moh.gov.lr/.

[85] Hughes J, Glassman A, Gwenigale W. Innovative Financing in Early Recovery: The Liberia Health Sector Pool Fund. 2012 [cited 2025 Apr 8]; Available from: www.cgdev.org.

[86] Nyenswah TG, Kateh F, Bawo L, Massaquoi M, Gbanyan M, Fallah M, et al. Ebola and its control in Liberia, 2014–2015. Emerg Infect Dis. 2016;22(2):169. 26811980 10.3201/eid2202.151456 PMC4734504

[87] Fall IS. Ebola virus disease outbreak in Liberia: application of lessons learnt to disease surveillance and control. Pan Afr Med J. 2019;33(Suppl 2):1. 31448046 10.11604/pamj.supp.2019.33.2.19074 PMC6691603

[88] Saeedzai SA, Blanchet K, Alwan A, Safi N, Salehi A, Singh NS, et al. Lessons from the development process of the Afghanistan integrated package of essential health services. BMJ Glob Health. 2023;8(9):12508. 10.1136/bmjgh-2023-012508 PMC10546159 37775105

[89] Saeed KMA, Osmani S, Collins D. Calculating the cost and financing needs of the basic package of health services in Afghanistan: methods, experiences, and results. Glob Health Sci Pract. 2022;10(4):e2100658. 36041844 10.9745/GHSP-D-21-00658 PMC9426985

[90] Mobilizing NGOs through Coordinated Donor and Ministry Support for Basic Health Care Service Delivery in Afghanistan, 2002–14 CASE STUDY.

[91] Cockcroft A, Khan A, Md Ansari N, Omer K, Hamel C, Andersson N. Does contracting of health care in Afghanistan work? Public and service-users’ perceptions and experience. BMC Health Serv Res. 2011;11(Suppl 2):S11. 22376191 10.1186/1472-6963-11-S2-S11 PMC3332555

[92] Afghanistan: Aid Cutbacks, Taliban Abuses Imperil Health | Human Rights Watch. [cited 2025 Apr 8]. Available from: https://www.hrw.org/news/2024/02/12/afghanistan-aid-cutbacks-taliban-abuses-imperil-health.

[93] Afghanistan’s health system suffers critical underfunding, calls for donor support. [cited 2025 Apr 8]. Available from: https://www.who.int/news/item/18-08-2023-afghanistan-s-health-system-suffers-critical-underfunding--calls-for-donor-support.

[94] Boland ST, Balabanova D, Mayhew S. Examining the militarised hierarchy of Sierra Leone’s Ebola response and implications for decision making during public health emergencies. Glob Health. 2023;19(1):1–23. 10.1186/s12992-023-00995-w. 10.1186/s12992-023-00995-w PMC10664671 37993942

[95] Hubmann M. Chronicity of disruptive project rhythms: the projectification of the ‘post-Ebola’’ health system rebuilding in Sierra Leone’. Time Soc. 2021;30(3):379–401. 10.1177/0961463X211005207.

[96] Barr A, Garrett L, Marten R, Kadandale S. Health sector fragmentation: three examples from Sierra Leone 16 studies in human society 1605 policy and administration. Glob Health. 2019;15(1):1–8. 10.1186/s12992-018-0447-5. 10.1186/s12992-018-0447-5 PMC6341573 30670026

[97] Somalia’s Famines, Government Apathy and the Aid Industry-the Elephant. [cited 2025 Apr 15]. Available from: https://www.theelephant.info/analysis/2022/01/28/somalias-famines-government-apathy-and-the-aid-industry/.

[98] Morrison J, Malik SMMR. Population health trends and disease profile in Somalia 1990–2019, and projection to 2030: will the country achieve sustainable development goals 2 and 3? BMC Public Health. 2023;23(1):1–9. 10.1186/s12889-022-14960-6. 36627611 10.1186/s12889-022-14960-6 PMC9832660

[99] Menkhaus K. Aid and institution-building in fragile states: The case of Somali-inhabited Eastern Horn of Africa. 2014 [cited 2025 Apr 16];2014. Available from: https://www.wider.unu.edu/node/721.

[100] Bile K, Warsame M, Ahmed AD. Fragile states need essential national health research: the case of Somalia. Lancet Glob Health. 2022;10(5):e617–8. 35427511 10.1016/S2214-109X(22)00122-X

[101] Murid Partaw A, Demerew K. Institutional logic of fragile states: Afghanistan and South Sudan in comparative perspective. J Middle East Afr. 2024;15(4):371–95.

[102] Jailobaeva K, Falconer J, Loffreda G, Arakelyan S, Witter S, Ager A. An analysis of policy and funding priorities of global actors regarding noncommunicable disease in low- and middle-income countries. Glob Health. 2021;17(1):1–15. 10.1186/s12992-021-00713-4. 10.1186/s12992-021-00713-4 PMC8240078 34187499

[103] Ibrahim M, Rizwan H, Afzal M, Malik MR. Mental health crisis in Somalia: a review and a way forward. Int J Ment Health Syst. 2022;16(1):1–12. 10.1186/s13033-022-00525-y. 35139873 10.1186/s13033-022-00525-y PMC8827242

[104] Governance and Health in Post-Conflict Countries: The Ebola Outbreak in Liberia and Sierra Leone - International Peace Institute. [cited 2025 Apr 17]. Available from: https://www.ipinst.org/2016/06/ebola-outbreak-liberia-sierra-leone.

[105] Squire JS, Hann K, Denisiuk O, Zachariah R. Staffing in public health facilities after the Ebola outbreak in rural Sierra Leone: how much has changed? F1000Res. 2020;8:793. 10.12688/f1000research.18566.1 PMC7047906 32148756

[106] Kangbai JB, Sesay U, Kangbai DM, Kagbanda FK. Public health system in post-pandemic Sierra Leone: a scoping review. BMC Infect Dis. 2024;24(1):1–13. 10.1186/s12879-024-10360-w. 39709377 10.1186/s12879-024-10360-w PMC11662533

[107] Barr A, Garrett L, Marten R, Kadandale S. Health sector fragmentation: three examples from Sierra Leone. Glob Health. 2019;15(1):8. 10.1186/s12992-018-0447-5 PMC6341573 30670026

[108] Renewing Focus on Effective Aid: Achieving the Vision of the Paris Declaration. [cited 2025 Apr 17]. Available from: https://blogs.worldbank.org/en/dev4peace/renewing-focus-on-effective-aid--achieving-the-vision-of-the-paris-declaration.

[109] Durrance-Bagale A, Marzouk M, Tung LS, Agarwal S, Aribou ZM, Ibrahim NBM, et al. Community engagement in health systems interventions and research in conflict-affected countries: a scoping review of approaches. Glob Health Action. 2022. 10.1080/16549716.2022.2074131. 35762841 10.1080/16549716.2022.2074131 PMC9246261

[110] Allotey P, Tan DT, Kirby T, Tan LH. Community engagement in support of moving toward universal health coverage. Health Syst Reform. 2019;5(1):66–77. 10.1080/23288604.2018.1541497. 30924744 10.1080/23288604.2018.1541497

[111] Issak E, Stella Silas K. Monitoring and evaluation systems on performance of community health workers programme in Banadir Region, Somalia. 2023. Int J Sci Res (IJSR). 10.21275/SR231029123759.

[112] Yassin sheikh Ali A, Khadar Abdi Jama A. Determinants of Islamic insurance acceptance: empirical evidence from Somalia. 2016 [cited 2025 Sep 15]; Available from: https://www.researchgate.net/publication/303700695.

